# Clinical Course of TGA After Arterial Switch Operation in the Current Era

**DOI:** 10.1016/j.jacadv.2023.100772

**Published:** 2023-12-27

**Authors:** Leo J. Engele, Roel L.F. van der Palen, Renée S. Joosen, Gertjan T. Sieswerda, Paul H. Schoof, Joost P. van Melle, Rolf M.F. Berger, Ryan E. Accord, Lukas A.J. Rammeloo, Thelma C. Konings, Wim A. Helbing, Jolien W. Roos-Hesselink, Pieter C. van de Woestijne, Stefan Frerich, Arie P.J. van Dijk, Irene M. Kuipers, Mark G.H. Hazekamp, Barbara J.M. Mulder, Johannes M.P.J. Breur, Nico Blom, Monique R.M. Jongbloed, Berto J. Bouma

**Affiliations:** a Department of Cardiology, Center for Congenital Heart Disease Amsterdam-Leiden (CAHAL), Amsterdam UMC, University of Amsterdam, Amsterdam, the Netherlands; b Netherlands Heart Institute, the Netherlands; c Division of Pediatric Cardiology, Department of Pediatrics, Center for Congenital Heart Disease Amsterdam-Leiden (CAHAL), Leiden University Medical Center, Leiden, the Netherlands; d Wilhelmina Children’s Hospital, University Medical Centre Utrecht, Utrecht, the Netherlands; e Department of Cardiology, University Medical Center Utrecht, Utrecht, the Netherlands; f Department of Pediatric Cardiac Surgery, Wilhelmina Children’s Hospital (Part of University Medical Center Utrecht), Utrecht, the Netherlands; g Department of Cardiology, University Medical Center Groningen, Groningen, the Netherlands; h Department of Pediatric Cardiology, Centre for Congenital Heart Diseases, Beatrix Children’s Hospital, University Medical Center Groningen, University of Groningen, Groningen, the Netherlands; i Department of Cardiothoracic Surgery, University Medical Center Groningen, Groningen, the Netherlands; j Division of Pediatric Cardiology, Department of Pediatrics, Center for Congenital Heart Disease Amsterdam-Leiden (CAHAL), Amsterdam UMC, Vrije Universiteit Amsterdam, Amsterdam, the Netherlands; k Department of Cardiology, Center for Congenital Heart Disease Amsterdam-Leiden (CAHAL), Amsterdam UMC, Vrije Universiteit Amsterdam, Amsterdam, the Netherlands; l Department of Pediatric Cardiology, Erasmus Medical Center, Rotterdam, the Netherlands; m Department of Cardiology, Erasmus Medical Center, Rotterdam, the Netherlands; n Department of Cardiothoracic Surgery, Erasmus Medical Center, Rotterdam, the Netherlands; o Department of Pediatric Cardiology, Academic Hospital Maastricht, Maastricht, the Netherlands; p Department of Pediatric Cardiology, Radboud University Medical Center, Nijmegen, the Netherlands; q Department of Cardiology, Radboud University Medical Center, Nijmegen, the Netherlands; r Division of Pediatric Cardiology, Department of Pediatrics, Center for Congenital Heart Disease Amsterdam-Leiden (CAHAL), Amsterdam UMC, University of Amsterdam, Amsterdam, the Netherlands; s Department of Cardiothoracic Surgery, Center for Congenital Heart Disease Amsterdam-Leiden (CAHAL), Leiden University Medical Center, Leiden, the Netherlands; t Department of Cardiology, Center for Congenital Heart Disease Amsterdam-Leiden (CAHAL), Leiden University Medical Center, Leiden, the Netherlands; u Department of Anatomy and Embryology, Center for Congenital Heart Disease Amsterdam-Leiden (CAHAL), Leiden University Medical Center, Leiden, the Netherlands

**Keywords:** arterial switch operation, long-term outcome, re-intervention, transposition of the great arteries

## Abstract

**Background:**

The number of patients with an arterial switch operation (ASO) for transposition of the great arteries (TGA) is steadily growing; limited information is available regarding the clinical course in the current era.

**Objectives:**

The purpose was to describe clinical outcome late after ASO in a national cohort, including survival, rates of (re-)interventions, and clinical events.

**Methods:**

A total of 1,061 TGA-ASO patients (median age 10.7 years [IQR: 2.0-18.2 years]) from a nationwide prospective registry with a median follow-up of 8.0 years (IQR: 5.4-8.8 years) were included. Using an analysis with age as the primary time scale, cumulative incidence of survival, (re)interventions, and clinical events were determined.

**Results:**

At the age of 35 years, late survival was 93% (95% CI: 88%-98%). The cumulative re-intervention rate at the right ventricular outflow tract and pulmonary branches was 36% (95% CI: 31%-41%). Other cumulative re-intervention rates at 35 years were on the left ventricular outflow tract (neo-aortic root and valve) 16% (95% CI: 10%-22%), aortic arch 9% (95% CI: 5%-13%), and coronary arteries 3% (95% CI: 1%-6%). Furthermore, 11% (95% CI: 6%-16%) of the patients required electrophysiological interventions. Clinical events, including heart failure, endocarditis, and myocardial infarction occurred in 8% (95% CI: 5%-11%). Independent risk factors for any (re-)intervention were TGA morphological subtype (Taussig-Bing double outlet right ventricle [HR: 4.9, 95% CI: 2.9-8.1]) and previous pulmonary artery banding (HR: 1.6, 95% CI: 1.0-2.2).

**Conclusions:**

TGA-ASO patients have an excellent survival. However, their clinical course is characterized by an ongoing need for (re-)interventions, especially on the right ventricular outflow tract and the left ventricular outflow tract indicating a strict lifelong surveillance, also in adulthood.

Dextro-transposition of the great arteries (TGA) is a complex cyanotic congenital malformation and represents approximately 5% to 7% of all congenital heart defects.^1^ Nowadays, surgical repair is performed several days after birth by the arterial switch operation (ASO). The ASO was introduced by Jatene et al^2^ in 1975 and involves translocation of the great arteries and reimplantation of the coronary arteries to achieve anatomical correction of the circulation. Despite the anatomical repair, complications during follow-up after ASO are common including obstructions in the right ventricular outflow tract (RVOT) and pulmonary branch stenosis due to the LeCompte maneuver, neo-aortic valve regurgitation due to neo-aortic root dilatation and coronary artery obstruction, although reported with various incidences.^3^, ^4^, ^5^ Previous studies on survival, (re)interventions, and clinical events after ASO are often limited because of their retrospective nature, small cohort size and by a possible bias due to an excess of events in patients operated in the early years with lack of experience of the surgical team. Surgical modifications have been applied since the first ASO including Lecompte maneuver, coronary reimplantation technique, and RVOT reconstruction. Whether clinical outcomes in the early decades after ASO are still applicable to nowadays care in the 21st century is unknown. Therefore, we used prospectively collected data from a nationwide registry from the current era to investigate the clinical course of patients with TGA after ASO, especially for the survival, the need for (re-)interventions and clinical events.

## Methods

### Study population

All patients diagnosed with TGA including the various subtypes, TGA with intact ventricular septum (TGA-IVS), TGA with ventricular septal defect (TGA-VSD), and Taussig-Bing double outlet right ventricle (TB-DORV) with subpulmonary VSD were included. Prospectively collected data were obtained from: 1) a national registry for pediatric patients with congenital heart disease (KinCor registry)^6^; and 2) a national registry for adult patients with Congenital Corvitia (CONCOR registry).^7^ Informed consent was obtained from all Kincor or Concor patients. The study was approved by the Medical Ethical Committee of the Amsterdam and Leiden University Medical Center and complies with the Declaration of Helsinki.

### Data collection

A query was performed to identify all TGA patients after ASO from the KinCor and Concor registry. The number of included patients in the registries corresponds to 75% to 80% of eligible patients and with 95 % of all the patients who were asked for participation.^6^ Patients were excluded based on the following criteria; prior surgical repair before ASO (eg, atrial switch) and lost to follow-up (no available follow-up data after inclusion). Baseline characteristics at the time of inclusion and all prospectively follow-up data (at least 30 days after ASO) were collected (period February 2001-July 2022). Follow-up ended at the most recent outpatient clinic contact. Patients were censored at the time of follow-up. Duplicated patients between the pediatric and adult database were identified based on diagnosis, ASO date, center and sex and subsequently removed. Quality checks were performed and consisted of identifying incomplete or inconsistent information and were resolved on a per-patient basis.

### Endpoints

Demographics and follow-up data, including reoperations, catheter-based interventions, and clinical events were analyzed. We aimed to describe the long-term outcome without early postoperative complications, therefore only clinical events, mortality, and re-interventions (surgery and catheter-based interventions) at least 30 days after ASO were included. Patients who died within 30 days after ASO were excluded from the analysis. Based on the localization, re-interventions were classified in the following categories: 1) RVOT, including re-interventions at the level of the pulmonary branches, main pulmonary artery, pulmonary valve or subpulmonary valve; 2) left ventricular outflow tract (LVOT), including re-interventions at the neo-aortic valve and neo-aortic root; 3) coronary arteries including any coronary revascularization procedure; 4) aortic arch; and 5) miscellaneous, including re-interventions at the mitral and tricuspid valve, atrial or ventricular septum. Electrophysiological interventions were included separately and consisted of catheter-ablation for supraventricular or ventricular arrhythmia and implantation of an implantable cardioverter-defibrillator (ICD) or pacemaker.

The following clinical events were included: endocarditis, myocardial infarction, heart failure, cardiac arrest, and late mortality. Myocardial infarction was defined as hospital admission for the presence of ST-segment elevation due to an acute coronary syndrome or patients with coronary occlusion with fibrotic scar or perfusion defect in the corresponding area. Heart failure episode was defined as the need for hospitalization for heart failure and assessed primary by the treating physician or by research nurse. Patients without any available follow-up data were considered as lost to follow-up. Only late mortality (>30 days after ASO) was included and classified when available.

### Statistical analysis

Data were summarized as number (%) for categorical variables and mean ± SD for continuous variables with normal distribution. Continuous data with skewed distribution were described by median (IQR). Baseline comparisons between TGA-IVS, TGA-VSD, and TB-DORV were performed by 1-way analysis of variance, Kruskal-Wallis tests, or chi-squared test, where appropriate. Factors associated with (re-)intervention were explored in univariable Cox regression analysis and only variables significant in univariable analysis were analyzed in multivariable Cox regression. For time-to-event analyses, patient-time was accrued until the outcome of interest (RVOT re-intervention, LVOT re-intervention, coronary intervention, electrophysiological intervention, and clinical event) with right censoring at the moment of the last follow-up. Cumulative incidence rates of (re-)interventions and clinical events with age as a time scale were adjusted for the competing risk of death and estimated for first occurrence of each outcome using a delayed entry method (R package survival) for left truncated data. Spline interpolation was used to report on the cumulative incidence estimates at the age of 40 years. Statistical analyses were performed using SPSS V.20 (IBM) and R v.4.0.3. (RStudio). Values of *P* < 0.05 were considered to be statistically significant.

## Results

### Patients

A total of 1,117 TGA-ASO patients were identified from the Kincor and Concor registry (Supplemental Table 1). Forty-three (3.7%) patients were lost to follow-up because they lived abroad (n = 21) or for reasons that are unknown (n = 22). Nine (0.8%) patients who died within 30 days after ASO and 4 (0.4%) patients who underwent atrial switch procedure prior to ASO were excluded. We included 1,061 patients, which were actively followed in 8 tertiary hospitals in the Netherlands. ASO was performed in 4 university medical centers. The diagnoses of patients included 696 (66%) TGA-IVS, 307 (29%) TGA-VSD, and 58 (5%) with TB-DORV. The median age at inclusion was 10.7 (IQR: 2.0-18.2, range 0-43) years. Pulmonary artery banding (PAB) was performed in 6% of the patients to prepare for the switch operation and 32% of the patients did have a surgical atrial septectomy or balloon atrial septostomy (Rashkind) procedure prior to ASO. Aortic arch abnormalities were present in 91 (9%) patients. The median age at ASO was 9 days (IQR: 6-19 days). Table 1 summarizes all baseline characteristics.

**Table 1:** Baseline Characteristics

	All Patients (n = 1,061)	TGA-IVS (n = 696)	TGA-VSD (n = 307)	TB-DORV (n = 58)	*P* Value^a^
Male	734 (69.2%)	495 (71.1%)	197 (64.2%)	42 (72.4%)	0.084
Age, y	10.7 (2.0-18.2)	10.9 (2.6-18.2)	11.0 (2.0-18.1)	5.6 (0.3-14.9)	0.026
Coexisting findings					
Aortic arch abnormality	91 (8.6%)	18 (2.6%)	43 (14.0%)	30 (51.7%)	<0.001
Preoperative procedures					
Rashkind/atrial septectomy	336 (31.7%)	239 (34.3%)	85 (27.7%)	12 (20.7%)	0.021
PAB	67 (6.3%)	25 (3.6%)	34 (11.1%)	8 (13.8%)	<0.001
Modified Blalock-Taussig shunt^b^	29 (2.7%)	14 (2.0%)	14 (4.6%)	1 (1.7%)	0.066
Arterial switch operation					
Age, d	9 (6-19)	8 (5-13)	11 (7-37)	39 (12-82)	<0.001

Values are n (%) or median (IQR).

Clinical characteristics of the study population at baseline in accordance with TGA subtype.

IVS = intact ventricular septum; PAB = pulmonary artery banding; TB-DORV = Taussig Bing double outlet right ventricle; TGA = transposition of the great arteries; VSD = ventricular septum defect.

a *P* value represents the simple comparisons between groups (Fisher exact test, Kruskal-Wallis or 1-way ANOVA).

b Systemic to pulmonary artery shunt.

### Survival

**Figure 1:**
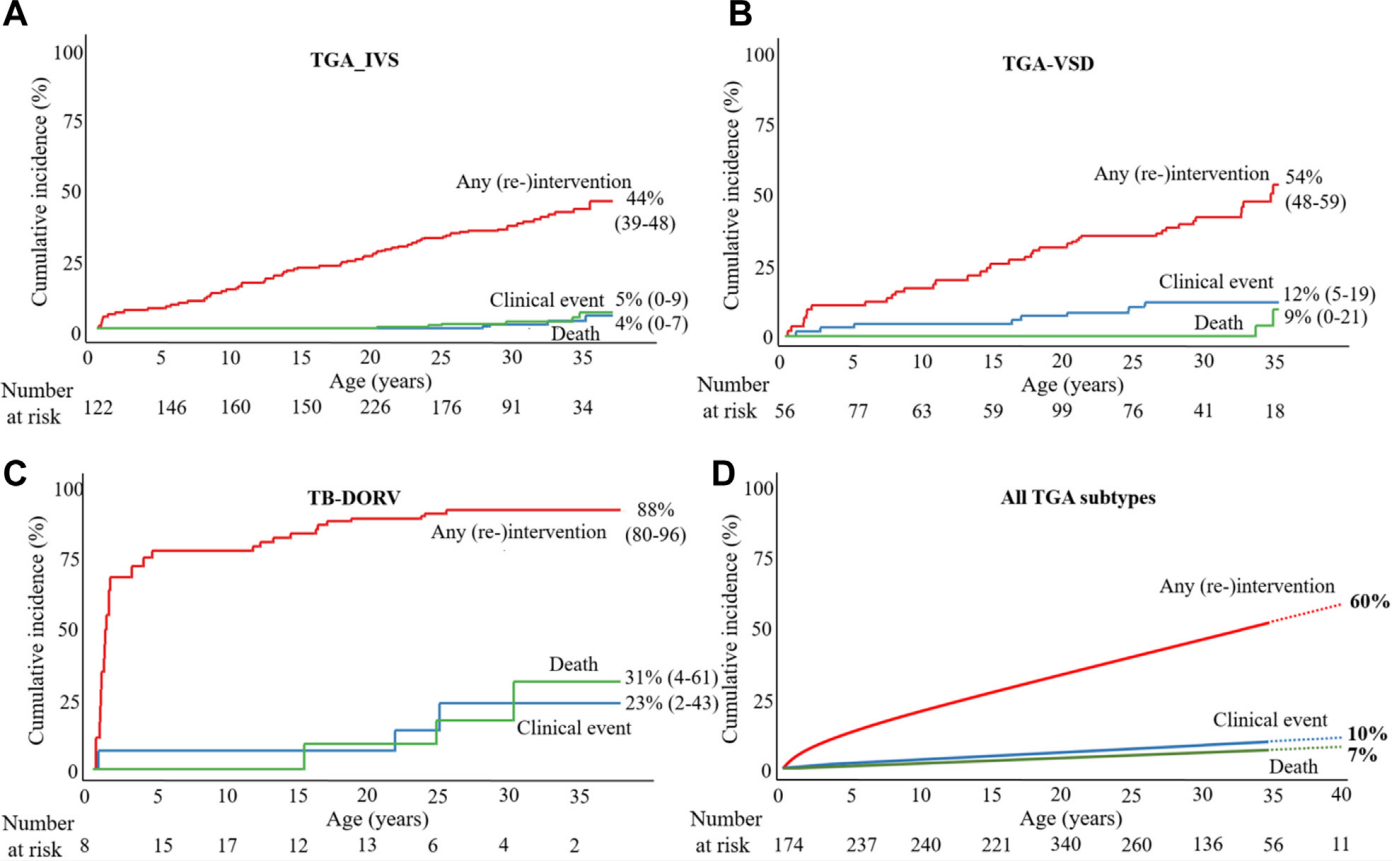
Cumulative Incidence of (Re-)Intervention, Clinical Event, and Death in TGA-ASO Cumulative incidence of first (re-)intervention during follow-up; all (re-)intervention (red), clinical event (blue) and death (green) in TGA-IVS (A), TGA-VSD (B), TB-DORV (C), all TGA subtypes (D). Cumulative incidence rates with age as a time scale were estimated for first occurrence of each outcome using a delayed entry method (R package survival) for left truncated data. (D) shows the cumulative incidence, calculated with spline interpolation, until the age of 40 years. ASO = arterial switch operation; IVS = intact ventricular septum; TB-DORV = Taussig Bing double outlet right ventricle; TGA = transposition of the great arteries; VSD = ventricular septum defect.

Patients were followed for a median period of 8.0 (IQR: 5.4-8.8, range 0-21) years. Forty-nine percent of the patients were older than 18 years at the end of follow-up. Twelve (1.1%) patients died during late follow-up (more than 30 days after surgery) at a median age of 23.1 years (IQR: 20.4-29.1 years). TGA subtypes in these patients were 6 (0.6%) TGA-IVS, 3 (0.3%) TGA-VSD, and 3 (0.3%) TB-DORV. Two patients (age 22 and 35 years) died due to progressive heart failure and in 1 patient (age 25 years) acute heart failure was the cause of death. Furthermore, 2 patients died after stent implantation in the pulmonary artery; in 1 patient (age 19 years) death was attributable to pulmonary artery embolism postprocedure and in the second patient (age 17 years) autopsy revealed an iatrogenic aorta pulmonary fistula. Infectious cause was present in 2 cases; 1 patient (age 28 years) died due to endocarditis of a Bentall prosthesis with severe neurological complications and the second patient (age 22 years) was diagnosed with lymphocytic myocarditis and died due to sudden cardiac death. One patient (age 2 months) passed away after cardiac arrest (ventricular fibrillation), but autopsy was not performed. In one 14-year-old patient, death was attributable to septic shock. Data on cause of death could not be retrieved in 3 patients. Late survival at 35 years was 95% (95% CI: 91%-100%) in TGA-IVS (Figure 1A), 91% (95% CI: 81%-100%) in TGA-VSD (Figure 1B), and 69% (95% CI: 39%-96%) in TB-DORV (Figure 1C). For the total cohort, survival at 35 years was 93% (95% CI: 88%-98%) (Figure 1D).

### (RE-)interventions

**Figure 2:**
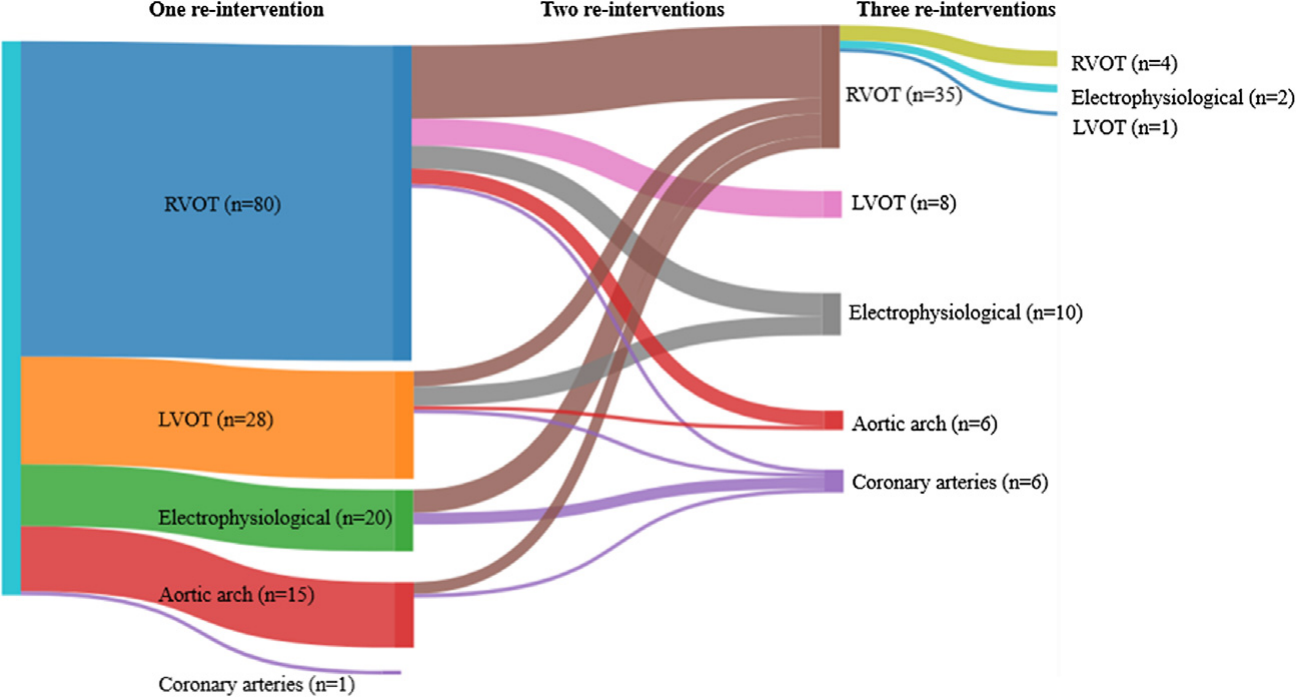
**Sankey Diagram of Patients With One or Multiple (Re-)Interventions After ASO** Sankey diagram: demonstrating the number of patients with single or multiple (re-)interventions after the arterial switch operation, categorized by the type of (re-)intervention. Electrophysiological (re-)interventions includes ablation, ICD and pacemaker placement. ASO = arterial switch operation; LVOT = left ventricular outflow tract; RVOT = right ventricular outflow tract.

In total, 240 (re-)interventions in 144 (13.6%) patients were performed during prospective follow-up, all (re-)interventions are listed in Table 2. Among TGA subgroups, the distribution of (re-)interventions was 76 (11%) in TGA-IVS, 42 (14%) in TGA-VSD, and 26 (45%) in TB-DORV. The median age at (re-)intervention was 17.8 (IQR: 9.3-22.5) years in TGA-IVS, 17.5 (IQR: 7.9-27.6) years in TGA-VSD, and 2.9 (IQR: 0.8-14.4) years in TB-DORV. Cumulative incidence of any (re-)intervention (n = 144) within the prospective follow-up interval 2001 to 2022 at the age of 35 years was 44% (95% CI: 39%-48%) in TGA-IVS (Figure 1A), 54% (95% CI: 48%-59%) in TGA-VSD (Figure 1B), and 88% (95% CI: 80%-96%) in TB-DORV (Figure 1C). The following univariable risk factors (Table 3) were significantly associated with any (re-)intervention post-ASO: morphological subtype (TB-DORV) and PAB. Independent risk factors included TB-DORV (HR: 4.9, 95% CI: 2.9-8.1, *P* < 0.001) and PAB (HR: 1.6, 95% CI: 1.0-2.2, *P* = 0.040) (Table 3 and Supplemental Figure 1). Figure 2 shows the number of patients with single or multiple (re-)interventions post-ASO.

**Table 2:** Overview of the Number of (Re-)Interventions in TGA-ASO During Prospective Follow-Up

	Total Re-Interventions	Re-Interventions in TGA-IVS (n = 696)	Re-Interventions in TGA-VSD (n = 307)	Re-Interventions in TB-DORV (n = 58)
Right ventricular outflow tract	**127**	**71**	**39**	**17**
Catheter-related intervention				
Dilatation or stent implantation	77	48	20	9
Surgical re-intervention				
Repair RVOT	38	17	13	8
Pulmonary valve replacement	12	6	6	0
Left ventricular outflow tract	**37**	**16**	**12**	**9**
Catheter-related intervention				
Neo-aortic root/valve	1	0	1	0
Surgical re-intervention				
Bentall procedure	19	9	7	3
Neo-aortic valve replacement	8	4	2	2
Neo-aortic valve plasty	5	1	1	3
Other^a^	4	2	1	1
Aortic arch	**21**	**1**	**9**	**11**
Catheter-related intervention				
Dilatation or stent implantation	11	0	2	9
Surgical re-intervention				
Aortic arch repair	10	1	7	2
Coronary arteries	**7**	**4**	**1**	**2**
Catheter-related intervention				
Dilatation or stent implantation	1	1	0	0
Surgical re-intervention				
Ostial plasty	2	0	1	1
CABG	4	3	0	1
Electrophysiological interventions	**32**	**14**	**13**	**5**
Ablation procedure	19	9	8	2
ICD placement	8	4	3	1
Pacemaker placement	5	1	2	2
Miscellaneous^b^	**16**	**3**	**9**	**4**

Values are n. **Bold** values represents the total number of re-interventions in each category.

ASO = arterial switch operation; CABG = coronary artery bypass grafting; DORV = double outlet right ventricle; ICD = implantable cardioverter defibrillator; IVS = intact ventricular septum; RVOT = right ventricular outflow tract; TB = Taussig Bing; TGA = transposition of the great arteries; VSD = ventricular septum defect.

a Personalized external aortic root support (Pears) (n = 3) and surgical relief of LVOT obstruction (n = 1).

b Mitral valve plasty (n = 7), ventricular septum defect closure (n = 6), tricuspid valve plasty (n = 2), and atrial septum defect closure (n = 1).

**Table 3:** Univariable and Multivariable Cox Proportional Hazard Analysis for (Re-)Intervention After ASO

Risk Factors	Univariable			Multivariable		
	HR	95% CI	*P* Value^a^	HR	95% CI	*P* Value^b^
Data source						
Concor registry	1.00					
KinCor registry	1.20	0.88-1.56	0.564			
Morphological subtype						
TGA-IVS	1.00			1.00		
TGA-VSD	0.92	0.62-1.36	0.673	1.17	0.78-1.80	0.441
TB-DORV	5.65	3.58-8.91	0.001	4.86	2.92-8.10	0.001
Sex						
Male	1.00					
Female	1.06	0.73-1.54	0.767			
Prior PA banding						
No	1.00			1.00		
Yes	1.66	1.24-2.19	0.006	1.60	1.02-2.21	0.040
Era of ASO (<2,000)						
No	1.00					
Yes	0.18	0.11-0.29	0.071			

ASO = arterial switch operation; IVS = intact ventricular septum; PA = pulmonary artery; TB-DORV = Taussig Bing double outlet right ventricle; TGA = transposition of the great arteries; VSD = ventricular septum defect.

a *P* value represents the *P* value for univariable Cox proportional hazards regression.

b *P* value for multivariable Cox proportional hazards regression.

### Right ventricular outflow tract

**Figure 3:**
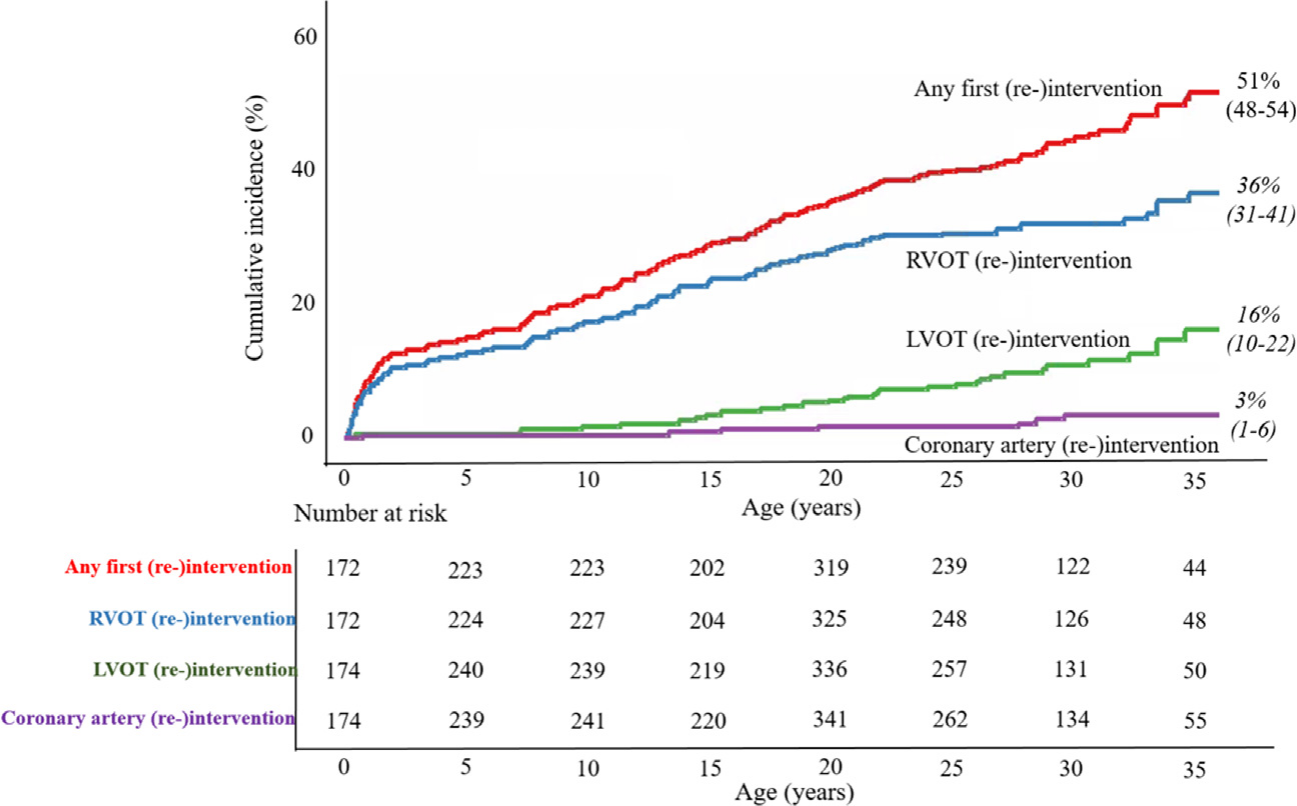
**Cumulative Incidence of (Re-)Intervention in TGA-ASO** Cumulative incidence of first re-intervention during follow-up; all (re-)intervention (red), RVOT re-intervention (blue), LVOT re-intervention (green), and coronary artery re-intervention (purple) in TGA patients after ASO. Cumulative incidence rates with age as a time scale were estimated for first occurrence of each outcome using a delayed entry method (R package survival) for left truncated data. ASO = arterial switch operation; LVOT = left ventricular outflow tract; RVOT = right ventricular outflow tract; TGA = transposition of the great arteries.

**Figure 4:**
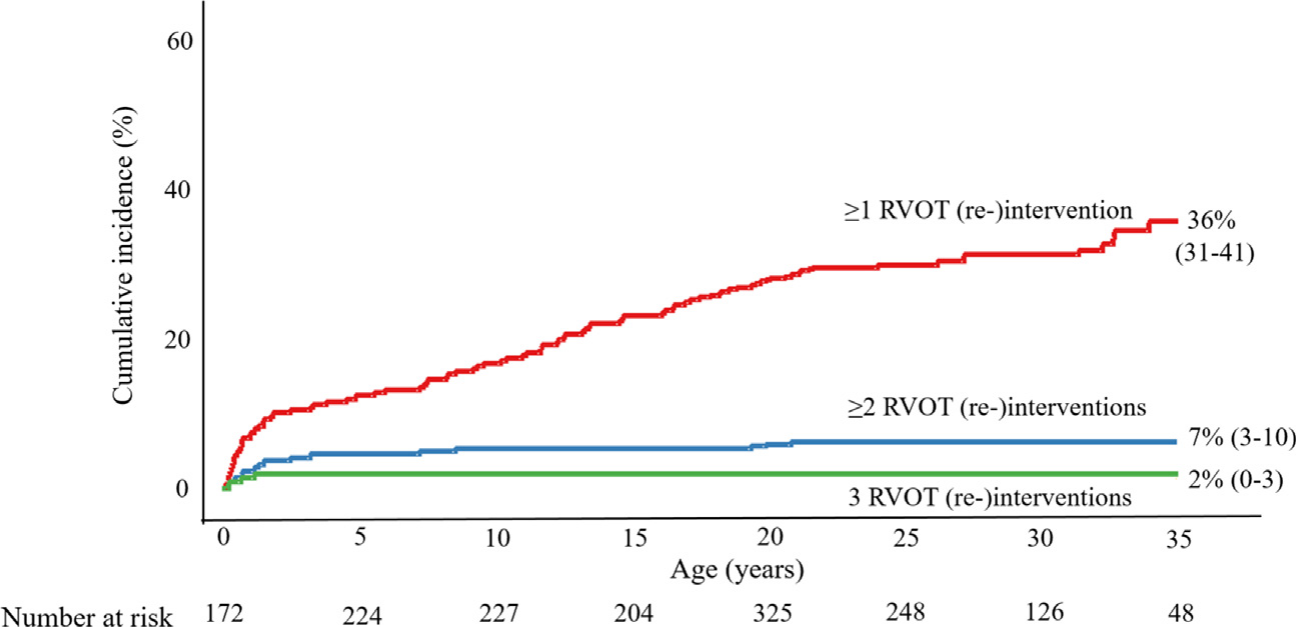
**Cumulative Incidence of First and Subsequently RVOT Re-Intervention in TGA-ASO** Cumulative incidence of first (red) and subsequently (blue and green) RVOT re-intervention within the follow-up period in TGA patients after ASO. Cumulative incidence rates with age as a time scale were estimated for each outcome using a delayed entry method (R package survival) for left truncated data. ASO = arterial switch operation; RVOT = right ventricular outflow tract; TGA = transposition of the great arteries.

A total number of 95 (9.0%) patients underwent 127 re-interventions at the site of the RVOT, consisting of 50 reoperations and 77 catheter-based procedures (Table 2). During prospective follow-up, RVOT re-interventions were performed in 55 (8%) TGA-IVS patients at the median age of 13.6 (IQR: 7.7-20.3) years, in 28 (9%) patients with TGA-VSD at the median age of 13.7 (IQR: 4.8-20.0) years, and in 12 (21%) patients with TB-DORV at the median age of 1.1 (IQR: 0.8-3.5) years. The cumulative incidence of RVOT re-intervention for all TGA subtypes at 10, 20, 30, and 35 years was, respectively, 17% (95% CI: 13%-21%), 28% (95% CI: 23%-31%), 32% (95% CI: 27%-36%), and 36% (95% CI: 31%-41%) (Figure 3) with a linear relationship between age and the cumulative RVOT re-intervention rate. Cumulative incidence in patients with ≥1 (n = 95), ≥2 (n = 20), or 3 (n = 4) consecutive RVOT re-interventions during follow-up was, respectively, 36% (95% CI: 31%-41%), 7% (95% CI: 3%-10%), and 2% (95% CI: 0%-2%) (Figure 4) at age 35 years. In patients with a complex morphological subtype (TB-DORV), the RVOT re-intervention rate was significantly higher (HR: 3.2, 95% CI: 1.7-6.4, *P* < 0.001).

### Left ventricular outflow tract

The number of re-interventions at the LVOT (neo-aortic root and neo-aortic valve) was 37 (3.5%), which were performed in 15 (2%) patients with TGA-IVS (median age 23.0 [IQR: 18.3-26.9] years), 14 (5%) patients with TGA-VSD (median age 26.3 [IQR: 16.7-28.9] years), and 8 (14%) patients with TB-DORV (median age 16.3 [IQR: 15.1-20.6] years. Most common neo-aortic re-interventions included Bentall procedures (n = 19) and neo-aortic valve replacements (n = 8) (Table 2). One patient underwent a catheter-related neo-aortic valve procedure. Cumulative incidence of LVOT re-intervention during follow-up was 6% (95% CI: 3%-9%) at 20 years, 10% (95% CI: 6%-14%) at 30 years, and 16% (95% CI: 10%-22%) at 35 years (Figure 3). On multivariable analysis, complex morphological subtype (TB-DORV) was an independent risk factor for LVOT re-intervention (HR: 6.1, 95% CI: 2.5-14.9, *P* < 0.001).

### Aortic arch

Re-intervention at the aortic arch was performed in 1 (0.1%) patient with TGA-IVS (median age 7.8), 9 (3%) patients with TGA-VSD (median age 6.9 [IQR: 3.1-13.4] years), and 11 (19%) patients with TB-DORV (median age 4.5 [IQR: 1.2-9.5] years), consisting of 11 catheter-related procedures (dilatation or stent placement) and 10 surgical procedures. The cumulative incidence was 9% (95% CI: 5%-13%) at 35 years. Independent risk factors for aortic arch re-intervention were aortic arch abnormality (HR: 3.0, 95% CI: 1.3-6.3, *P* < 0.001) and morphological subtype (TB-DORV) (HR: 4.2, 95% CI: 2.1-6.6, *P* < 0.001).

### Coronary arteries

During the follow-up period, 7 (0.7%) patients required a re-intervention at the coronary arteries at a median age of 17.0 years (IQR: 9.5-25.9 years). Four patients underwent coronary artery bypass grafting for coronary artery occlusion. All 4 patients showed signs of ischemia during exercise stress test or during cardiac stress imaging, however only 1 patient was symptomatic. Catheter-related coronary re-intervention for ostium stenosis of the left coronary artery was performed in 1 asymptomatic patient. Two patients underwent coronary plasty; 1 asymptomatic patient with functional occlusion of the left main and 1 symptomatic patient with stenosis of the left main. The cumulative incidence at 35 years was 3% (95% CI: 1%-6%). TGA subtype (TGA-VSD or TB-DORV) and surgical era (before 2000) were not significantly associated with coronary re-intervention in our cohort.

### Electrophysiological interventions

Nineteen (1.8%) patients underwent catheter ablation therapy for supraventricular (n = 11) or ventricular (n = 8) arrhythmia at a median age of 24.1 years (IQR: 20.5-28.4 years). Implantation of a pacemaker device for atrioventricular block was performed in 5 patients (0.5%) and 8 patients (0.8%) underwent ICD implantation because of ventricular arrhythmia. The cumulative incidence of any electrophysiological intervention was 4% (95% CI: 1%-6%) at 20 years and 11% (95% CI: 6%-16%) at 35 years.

### Clinical events

A total of 17 (1.6%) patients had at least 1 clinical event during follow-up, consisting of endocarditis (n = 8), heart failure (n = 8), myocardial infarction (n = 2), and cardiac arrest (n = 1). In 1 case, endocarditis led to the demise of the patient. Furthermore, 1 asymptomatic patient (age 31 years) was diagnosed with an old myocardial infarction and 1 symptomatic patient (age 27 years) presented with an acute coronary syndrome. Cumulative incidence of a clinical event at 35 years was 4% (95% CI: 0%-7%) in TGA-IVS (Figure 1A), 12% (95% CI: 5%-19%) in TGA-VSD (Figure 1B), and 23% (95% CI: 2%-43%) in TB-DORV (Figure 1C).

## Discussion

In this large nationwide prospective cohort study, consisting of 1,061 TGA patients after ASO, we estimated cumulative incidence rates for (re-)intervention and clinical event after ASO. This study reported a late survival of 93% at 35 years, showing that in the current era patients after ASO have an excellent long-term survival compared to a 64% survival at 40 years as assessed in a recently published meta-analysis of dextro-TGA patients corrected by the atrial switch.^8^ However, compared to the survival rate of 98.7% at the age of 35 years in the general Dutch population, survival after ASO is still lower. In contrast to all previous retrospective studies with inclusion of events from the early decades after ASO, our prospective analysis on outcome (with age as the primary time scale) in exclusively the 21st century gives in our view a more up to date estimate of the clinical course of TGA-ASO patients in the current era. In this study, we demonstrated that (re-)intervention rates appeared to be high and linear with age, the cumulative incidence of (re-)intervention at the age of 35 years was 52%. However, this rate is still lower compared to a morbidity rate of 81% at 39 years after atrial switch.^9^

### Methodological approach

In the current study, prospectively collected data from 2 nationwide registries with inclusion of pediatric and adult TGA-ASO patients were analyzed. Age was used as the primary time scale and allowed us to describe the clinical course of TGA-ASO patients regarding clinical events and (re-)interventions. With this approach, we did account for the confounding effect of age and information bias was avoided. Re-interventions or clinical events before inclusion were not part of the prospective analysis. Our results were rather comparable with previous reported outcome after ASO.^10^, ^11^, ^12^ Therefore, we are confident that our results fairly represent a contemporary cohort of TGA-ASO patients.

### RVOT re-interventions

In both pediatric and adult ASO survivors, RVOT re-intervention was the most frequent re-intervention. The cumulative incidence was 36% at the age of 35 years and showed a linear relationship between RVOT re-intervention rate and age indicating that the need for RVOT re-intervention after ASO is not limited to a certain time frame but continues during adulthood. Santens et al^13^ reported a re-intervention rate of 21% at 20 years in their retrospective long-term follow-up analysis, which is in contrast to the re-intervention rate of 28% at 20 years in our study. However, this higher rate may be explained by our definition of RVOT interventions, which was not restricted to re-interventions at the pulmonary arteries but also included pulmonary valve replacements. In our study, the median age of the first RVOT re-intervention was 13.8 years and is different than in other studies in which the age of first RVOT re-intervention varied between 9 months and 3.8 years,^14^, ^15^, ^16^ probably caused by the longer observation period and the continuation of the need for intervention at older age. Patients with complex TGA subtype (DORV) were at increased risk for RVOT re-intervention, indicating the impact of this complex morphology on the clinical course. No association was found between the era in which the ASO was performed and the incidence of RVOT re-intervention probably as a result of the ongoing interventions also in adulthood.

### LVOT re-interventions

In our study, the cumulative incidence of patients who required a re-intervention at the LVOT was 7% at the age of 25 years. This finding agrees with the results of a recent retrospective study by Fricke et al^10^ who reported a freedom from neo-aortic re-intervention of 92% at 25 years. As most of these patients underwent neo-aortic re-interventions during adulthood (median age 21.3 years), it is expected that the number of re-interventions will increase as the follow-up lengthens. Literature on the progression of aortic root dilatation during lifetime is contradictory. Schwartz et al^17^ observed no further increase of neo-aortic root diameter 10 years after ASO. However, more recent studies in both children and adults found no stabilization of neo-aortic growth and reported an average growth rate of the neo-aortic root of 0.63 mm/year.^18^ In multivariable analysis, PAB was independently associated with LVOT re-intervention, which could be explained by stenosis and distortion due to banding of the pulmonary artery which facilitate neo-aortic regurgitation. Furthermore, analysis between TGA subtypes showed that TB-DORV was associated with LVOT re-intervention but we did not find, in contrary to other studies, an association in TGA-VSD patients. In our study, the cumulative incidence at 35 years was 16%, which demonstrates the ongoing risk for aortic root dilatation and aortic valve regurgitation during lifetime. Close surveillance of the neo-aorta in patients with or without risk factors seems to be indicated.

### Coronary arteries

With a cumulative incidence of 3% at the age of 35 years, the number of late re-intervention at the coronary arteries appeared to be low. This low rate is in line with current literature on late coronary complications after ASO.^19^ Of the 7 patients who underwent coronary re-intervention, only 2 patients experienced complaints of angina pectoris. In 5 asymptomatic patients, detection of coronary abnormality was based on routine exercise stress testing or cardiac imaging. The absence of angina pectoris in patients with coronary abnormalities may be due to denervation caused by transection of the great arteries and reimplantation of the coronary arteries.^20^ In contrast to previous studies, we did not find a relationship between complex TGA-subtype and coronary re-interventions, which may be explained by the low number of late coronary re-interventions in our study. None of the coronary re-interventions or coronary events were associated with late mortality. Although the optimal follow-up strategy of the coronary arteries is a contentious issue, our findings of a low cumulative incidence of coronary events and re-interventions does not support an aggressive routinely strategy regarding the clinical evaluation of the coronary arteries.

### Clinical implications

The cumulative incidence of a first re-intervention after ASO was 60% at 40 years, demonstrating the ongoing need for re-interventions after ASO. Long-term outcome after the age of 40 years is still unknown, however based on current re-intervention rates it may be expected that the number of patients with a first (re-)intervention will raise as the ASO population ages. Based on an estimated re-intervention rate of 14.9% per decade with a linear relationship with age, one could speculate a re-intervention rate up to 90% at the age of 60 years. Therefore, regular follow-up with focus on both RVOT and LVOT seems necessary.

### Study Limitations

Surgical technique may impact the long-term outcome. As our patient population included a nationwide TGA-ASO cohort, encompassing patients from different centers, we could not account for differences in surgical approaches between centers and surgeons. Although this enhances the external validity. Furthermore, analysis was performed with data extracted from 2 different registries, including the Concor registry (inclusion of adult patients since 2001) and the KinCor registry (inclusion of pediatric patients since 2013). Procedures which were performed before the ASO differed significantly between the 2 data sources and could be explained by the inclusion of patients from both centuries. Current analysis was not adjusted for multiple testing; therefore, CIs should be interpreted with caution. Our risk factor analysis was restricted to the available patient characteristics from the national registries, for example, details on coronary anatomy or ventricular function could not be taken into account as this was not available in the registry database.

## Conclusions

Our study shows that TGA patients who survived the ASO have an excellent late survival of 93% at 35 years. However, a significant number of patients required re-intervention especially at the RVOT and LVOT during lifetime. The number of coronary re-interventions and coronary events appeared to be very low (Central Illustration). The ongoing need for (re-)intervention highlights that a strict lifelong surveillance, also in adulthood remains necessary.

PERSPECTIVES **COMPETENCY IN MEDICAL KNOWLEDGE:** TGA patients who survived the arterial switch operation have an excellent late survival (93%) at 35 years. However, a significant number of patients required re-intervention at the RVOT and LVOT during lifetime. The number of coronary re-interventions and coronary events appeared to be very low.

**TRANSLATIONAL OUTLOOK:** Based on current re-intervention rates, it may be expected that the number of patients with a first re-intervention will raise as the ASO population ages. Therefore, regular follow-up with focus on both RVOT and LVOT seems recommended.

**Central Illustration:**
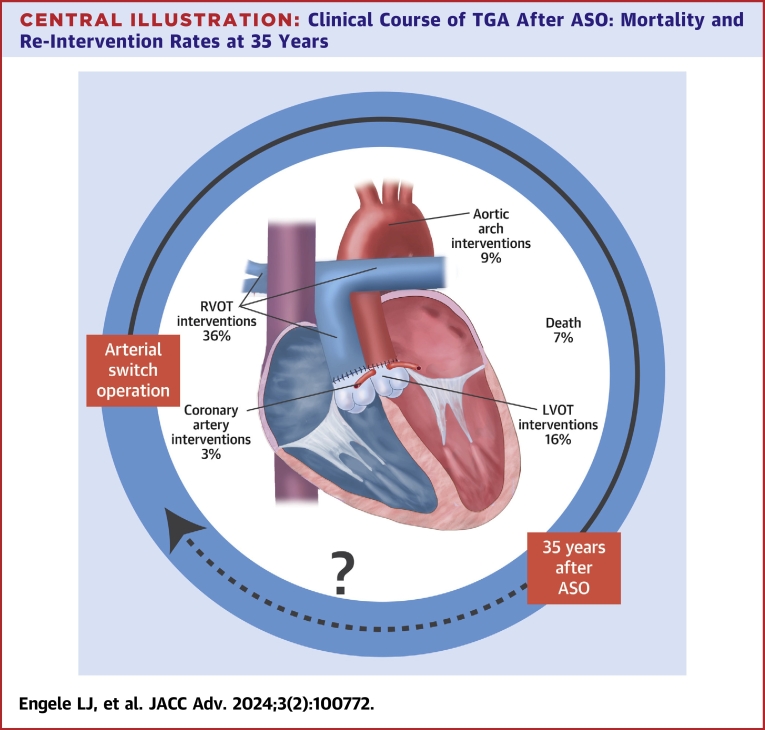
**Clinical Course of TGA After ASO: Mortality and Re-Intervention Rates at 35 Years** The figure demonstrates the clinical course of TGA after ASO at the age of 35 year. The mortality and re-intervention rates represents the calculated cumulative incidence at the age of 35 years for RVOT re-intervention, LVOT re-intervention, coronary artery re-intervention and death. The dotted line (and question mark) reflects the period after 35 years, in which the outcome is still unknown. ASO = arterial switch operation; LVOT = left ventricular outflow tract; RVOT = right ventricular outflow tract.

## Funding support and author disclosures

Support was received from the Netherlands Cardiovascular Research lnitiative: An initiative with support of the Dutch Heart Foundation and Hartekind, CVON2019-002 OUTREACH. The authors have reported that they have no relationships relevant to the contents of this paper to disclose.
