# Supplementary material for: Clinical Course of TGA After Arterial Switch Operation in the Current Era
Source: JACC Adv. 2023 Dec 27;3(2):100772. doi: 10.1016/j.jacadv.2023.100772 (PMC11198364; doi:10.1016/j.jacadv.2023.100772)
Supplement: Supplemental Figure 1 and Table 1 [file mmc1.docx]

Supplemental Appendix

| **Supplemental table 1.** Patient characteristics between KinCor and Concor registry | | | | |
| --- | --- | --- | --- | --- |
|  | All patients (n=1061) | KinCor (n=740) | Concor (n=321) | p-value |
| Male | 734 (69.2) | 514 (69.5) | 218 (67.9) | 0.664 |
| Age at inclusion, median (years) | 10.7 | 5.4 | 19.3 | <0.001 |
| TGA-IVS | 696 (65.6) | 477 (64.5) | 220 (68.5) | 0.071 |
| TGA-VSD | 307 (28.9) | 221 (29.9) | 86 (26.8) | 0.499 |
| TB-DORV | 58 (5.5) | 49 (6.6) | 9 (2.8) | 0.023 |
| Co-existing findings |  |  |  |  |
| - Aortic arch abnormality | 91 (8.6) | 68 (9.2) | 22 (6.9) | 0.312 |
| Preoperative procedures |  |  |  |  |
| - Raskind/atrial septectomy | 336 (31.7) | 258 (34.9) | 77 (24.0) | 0.002 |
| - PAB | 67 (6.3) | 33 (4.5) | 33 (10.3) | <0.001 |
| - Blalock procedure^b^ | 29 (2.7) | 9 (1.2) | 20 (6.2) | 0.066 |
| Arterial switch operation |  |  |  |  |
| - age, median (days) | 9 (6 - 19) | 10 (7 - 16) | 7 (2 - 43) | <0.001 |
| Values are n (%) or median (IQR). ^a^ P-value represents the simple comparisons between groups (Chi-squared test, Kruskal Wallis or One-way Anova, where appropriate). ^b^Systemic to pulmonary artery shunt. IVS = intact ventricular septum; PAB = pulmonary artery banding; TGA = transposition of the great arteries; TB-DORV = taussig bing double outlet right ventricle; VSD = ventricular septum defect. | | | | |

**Supplemental figure 1.** Independent risk factors for any (re-)intervention after ASO


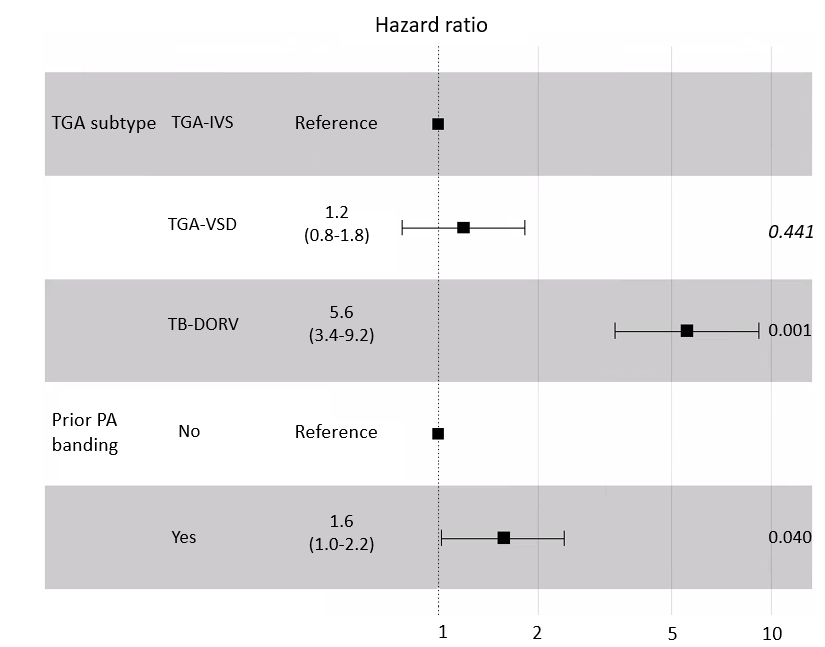


Multivariable cox proportional hazards regression analysis was used to identify risk factors for any (re-)intervention after ASO. IVS = intact ventricular septum; TGA = transposition of the great arteries; TB-DORV = taussig bing double outlet right ventricle; VSD = ventricular septum defect.
